# Optimization of Silicon Nitride Waveguide Platform for On-Chip Virus Detection

**DOI:** 10.3390/s22031152

**Published:** 2022-02-02

**Authors:** Raghi S. El Shamy, Mohamed A. Swillam, Xun Li

**Affiliations:** 1 Department of Electrical and Computer Engineering, Faculty of Engineering, McMaster University, Hamilton, ON L8S 4L8, Canada; elshamyr@mcmaster.ca; 2 Department of Physics, School of Science and Engineering, The American University in Cairo, New Cairo 11835, Egypt; m.swillam@aucegypt.edu

**Keywords:** virus detection, on-chip optical biosensors, Si_3_N_4_ waveguides

## Abstract

This work presents a rigorous and generic sensitivity analysis of silicon nitride on silicon dioxide strip waveguide for virus detection. In general, by functionalizing the waveguide surface with a specific antibodies layer, we make the optical sensor sensitive only to a particular virus. Unlike conventional virus detection methods such as polymerase chain reaction (PCR), integrated refractive index (RI) optical sensors offer cheap and mass-scale fabrication of compact devices for fast and straightforward detection with high sensitivity and selectivity. Our numerical analysis includes a wide range of wavelengths from visible to mid-infrared. We determined the strip waveguide’s single-mode dimensions and the optimum dimensions that maximize the sensitivity to the virus layer attached to its surface at each wavelength using finite difference eigenmode (FDE) solver. We also compared the strip waveguide with the widely used slot waveguide. Our theoretical study shows that silicon nitride strip waveguide working at lower wavelengths is the optimum choice for virus detection as it maximizes both the waveguide sensitivity (S_wg_) and the figure of merit (FOM) of the sensor. The optimized waveguides are well suited for a range of viruses with different sizes and refractive indices. Balanced Mach–Zehnder interferometer (MZI) sensors were designed using FDE solver and photonic circuit simulator at different wavelengths. The designed sensors show high FOM at λ = 450 nm ranging from 500 RIU^−1^ up to 1231 RIU^−1^ with L_MZI_ = 500 µm. Different MZI configurations were also studied and compared. Finally, edge coupling from the fiber to the sensor was designed, showing insertion loss (IL) at λ = 450 nm of 4.1 dB for the design with FOM = 500 RIU^−1^. The obtained coupling efficiencies are higher than recently proposed fiber couplers.

## 1. Introduction

The coronavirus disease pandemic of 2019 (COVID-19) is currently an exceptional threat to human lives all over the world. Its expeditious spread has led to millions of cases and hundreds of thousands of deaths in a few months. Almost all countries worldwide were forced to lockdown for several months to limit the spread of the virus, leading to devastating social and economic effects. In general, many people across the globe lose their life due to viral infection diseases [1]. Hence, simple, fast, cheap, and accurate detection of viruses are of great importance. Polymerase chain reaction (PCR) is one of the well-known methods used for virus detection and it is the primary method used currently for COVID-19 detection [2]. Although this technique is highly sensitive and accurate, it is expensive, time-consuming, and involves complex procedures and sample preparation.

Optical refractive index (RI) sensing is one of the main integrated optical techniques used for bio-detection [3,4,5,6,7,8]. RI sensors offer fast, compact, and cheap detection with high sensitivities. However, RI sensors are not selective as they only detect the change in the medium (clad) refractive index which can occur due to different substances. A widely used technique to solve this problem in bio-sensing is surface functionalization [9,10,11]. In surface functionalization, the surface of the sensing waveguide is coated with specific molecules called binder or capture molecules and immobilized through a certain process. These immobilized molecules selectively capture the analyte molecules to be detected from the whole sample. After sensor exposure to the sample, a washing step is needed to make sure that only the analyte of interest is present in the sensors’ medium (clad) hence, the detected refractive index change is due to this analyte alone.

RI sensors’ performance is determined mainly by the figure of merit (FOM) which is the ratio between the sensor sensitivity (S) and full width half maximum (FWHM) of the output spectrum, FOM = S/FWHM. RI sensors’ sensitivity (S) can be divided into device sensitivity (S_D_) and waveguide sensitivity (S_wg_). Device sensitivity is defined as the ratio between the change in the resonance wavelength and the change in the waveguide mode effective index, S_D_ = dλ_res_/dn_eff_. S_D_ is determined by the optical sensor configuration used and its dimensions such as Mach–Zhender interferometer and its arm’s length [3,4,5] or ring resonators and its ring radius [6,7,8]. While waveguide sensitivity is defined as the ratio between the change in the mode effective index and the change in the medium index, S_wg_ = dn_eff_/dn_med_. S_wg_ is determined by the sensing waveguide structure such as strip, rib, or slot waveguides and their dimensions. The overall sensitivity of the optical sensor is the product of both parameters, S = S_D_ × S_wg_. Hence, to maximize any RI sensor performance, waveguide sensitivity (S_wg_) should be maximized.

Silicon nitride on insulator (SiNOI) waveguide platform, where the insulator here is the silicon dioxide, offers numerous advantages for various applications [12,13,14,15]. Similar to silicon on insulator (SOI) platform SiNOI is a complementary metal-oxide semiconductor (CMOS) compatible allowing for mass-scale and low-cost fabrication [14,15]. It also allows for monolithic integration with silicon devices and other electronic circuitry [12]. The lower refractive index contrast of the SiNOI waveguide compared with SOI reduces scattering loss due to surface roughness resulting in much lower propagation losses [12,13,14], while still maintaining device compactness. This lower index contrast also makes SiNOI devices more tolerant to fabrication errors [12,13]. In addition, the Si_3_N_4_ thermo-optical coefficient is one order of magnitude lower than Si [13], hence Si_3_N_4_-based devices are less sensitive to temperature fluctuations.

Moreover, the SiNOI platform has a wider transparency range, from visible to mid-infrared, compared with the SOI platform [12,13,14,15]. This allows the realization of photonic applications outside the telecom bands, such as integrated optical phased arrays for LIDAR applications [16]. Finally, while silicon has a large Kerr effect, the two-photon absorption (TPA) prevents efficient nonlinear applications. Si_3_N_4_, on the other hand, has adequate Kerr nonlinearity and almost zero TPA [14,15]. Thus, the SiNOI platform allows for frequency comb as well as supercontinuum generation [17,18], which are essential for high data-rate telecommunications, high-resolution spectroscopy, and frequency metrology [19].

In this work, we present a detailed theoretical study and optimization of silicon nitride (Si_3_N_4_) on silicon dioxide (SiO_2_) waveguide platform for virus detection. The waveguide surface is assumed to be functionalized by the antibodies of the virus to be detected, using a process similar to that in [9,10,11] such that the medium index change is only due to this virus. A finite difference eigenmode (FDE) solver [20] is used to determine the waveguide dimensions that maximize the waveguide sensitivity (S_wg_) to a virus layer attached to its surface. Both fundamental quasi-transverse electric (TE) and quasi-transverse magnetic (TM) modes are studied. Moreover, slot waveguide was also analyzed and compared with the strip waveguide. Different operating wavelengths were examined from the visible to the mid-infrared range. We found that S_wg_ and FOM increase at lower wavelengths. This numerical analysis is essential to construct a cheap, mass-scale fabrication of a compact and highly sensitive RI optical sensor for fast virus detection and generally any biomolecule. The optimized waveguides can be used in different integrated optical devices such as interferometers and resonators to construct the virus sensor. MZI sensors utilizing the optimized waveguides were designed reaching FOM = 1231 RIU^−1^ at λ = 450 nm with 500 µm arms’ length. We also designed an MZI sensor with waveguide widths above 1 µm that can be easily fabricated in simple and cheap facilities. Finally, fiber edge coupling to the sensors chip was studied and optimized, showing higher coupling efficiencies than recently demonstrated fiber couplers. The analysis and results obtained here are generic, i.e., they can be applied to a wide range of biomolecules with different sizes and refractive indices.

## 2. Virus Sensing Waveguide Analysis and Discussion

**Figure 1:**
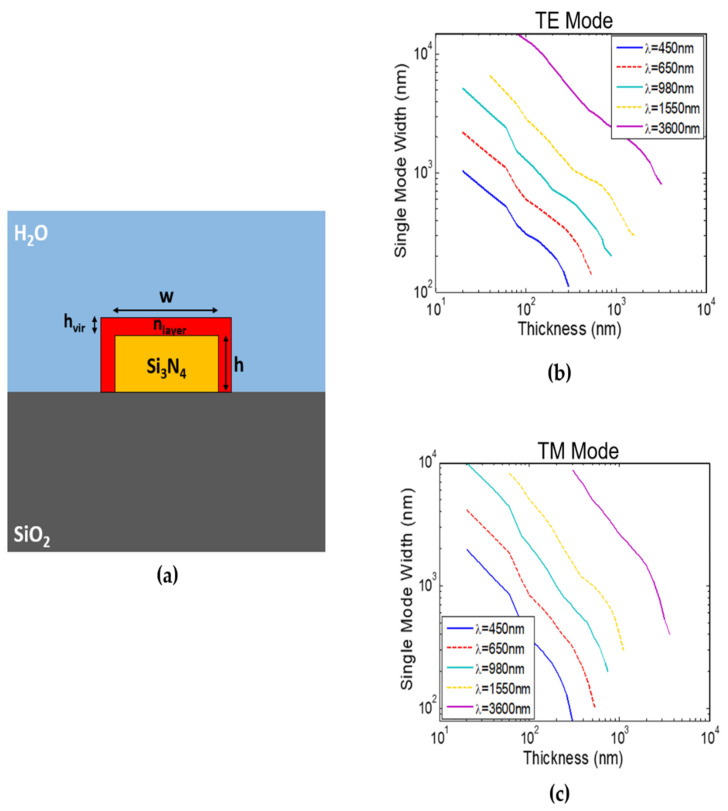
(**a**) Schematic of surface-functionalized Si_3_N_4_ on SiO_2_ strip waveguide for virus detection with a layer representing virus attachment. Strip waveguide single-mode width versus waveguide thickness at different wavelengths of: (**b**) TE mode and (**c**) TM mode.

Figure 1a shows the Si_3_N_4_ strip waveguide proposed for virus detection with width (w) and thickness (h) on SiO_2_ substrate and water clad. The waveguide surface is functionalized for selective detection such that only the virus of interest will adhere to the surface and form a layer. More details about the exact functionalization process can be found in [9,10,11]. Hence, we model this layer by a thickness h_vir_ equal to the virus diameter d_vir_, i.e., h_vir_ = d_vir_, and a refractive index n_layer_, as shown in Figure 1a. The refractive index n_layer_ is given by Equation (1). The value of n_layer_ changes between the refractive index of the water n_water_ and the refractive index of the virus n_vir_ according to the virus coverage fraction r.

(1) $$n_{\mathrm{layer}}=(1-r)\times n_{\mathrm{water}}+r\times n_{\mathrm{vir}}$$

In this case, virus binding to the immobilized antibodies on the waveguide surface will change this layer refractive index which will accordingly change the waveguide mode effective index. We choose h_vir_ = 80 nm which is in the range of the reported diameters for the COVID-19 virus [21,22]. All the results obtained here are using h_vir_ = 80 nm unless mentioned otherwise. This diameter is relatively small when compared with other viruses. We will see by the end of this section that for the same waveguide dimensions increasing the virus layer thickness (targeting virus diameter) will result in higher waveguide sensitivity S_wg_. Thus, the optimized waveguides at h_vir_ = 80 nm can be used for a range of viruses with different h_vir_ = d_vir_.

Different operating wavelengths are studied from visible range λ = 450 nm (blue) and λ = 650 nm (red), to near-infrared λ = 980 nm and λ = 1550 nm, and MIR λ = 3600 nm. Material dispersion is considered where silicon nitride, silicon dioxide, and water refractive index data along the wavelength are obtained from [23,24]. At each operating wavelength, we firstly define the single mode dimensions by determining the maximum width at different thicknesses using the FDE solver [20], as shown in Figure 1b,c. Then, we calculate the waveguide sensitivity (S_wg_) at different waveguide dimensions (w and h), for both fundamental quasi-TE and fundamental quasi-TM modes, which we will denote as TE and TM for simplicity. Note that, unlike most RI sensors designs, here we calculate the surface waveguide sensitivity, S_wg_ = dn_eff_/dn_layer_, not the bulk sensitivity, S_wg_ = dn_eff_/dn_clad_, which is more accurate for viral detection. S_wg_ is calculated with n_layer_ around the water index with Δn_layer_ = 0.001, which means that the waveguides are optimized to have maximum sensitivity when the virus layer index is changed slightly from that of water. This corresponds to a minimum virus coverage (r close to 0). Accordingly, the exact virus refractive index n_vir_ does not affect the obtained results. This makes our analysis independent of the virus refractive index and hence we do not need to have its value.

**Figure 2:**
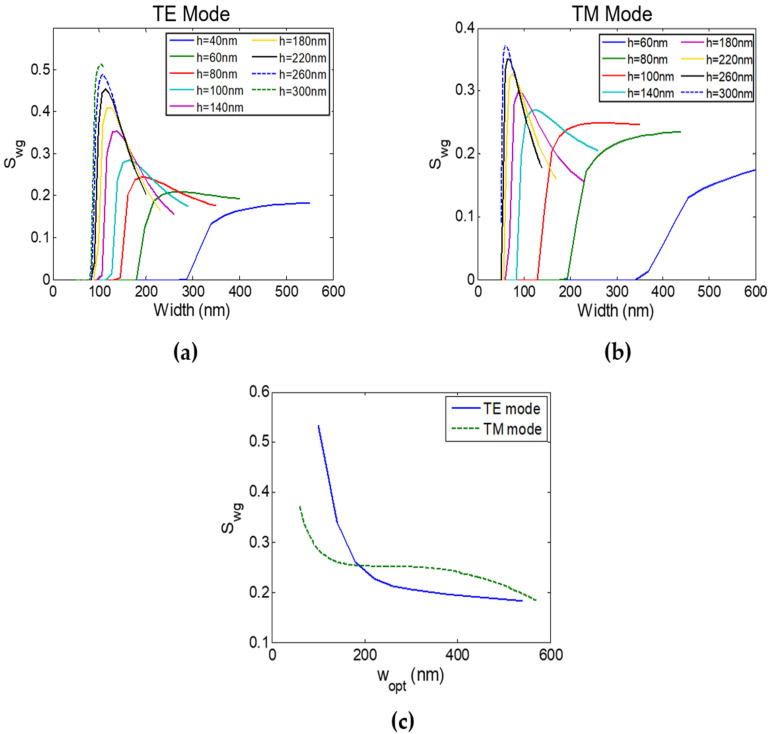
Strip waveguide sensitivity (S_wg_) versus waveguide width (w) at different thicknesses (h) at λ = 450 nm of: (**a**) TE mode and (**b**) TM mode. (**c**) Strip waveguide sensitivity (S_wg_) of both TE and TM modes versus the optimum waveguide width (w_opt_) at λ = 450 nm.

Figure 2a,b show the waveguide sensitivity of the TE mode and TM mode, respectively, for different widths and thicknesses at λ = 450 nm. Results show that for each waveguide thickness, there is an optimum width that maximizes the waveguide sensitivity. Such behavior is expected [25]. For large waveguide widths, most of the mode field is confined inside the silicon nitride core, resulting in low sensitivity. As the width decreases, the mode becomes less confined and the evanescent field in the cladding increases, increasing the sensitivity. However, for small widths, near cut-off, more field moves to the higher (than clad) refractive index substrate which again decreases the sensitivity [25]. Results also show that as the waveguide thickness increases, the optimum width (w_opt_) decreases, and optimum sensitivity increases, which is also expected [25]. Hence, waveguides with a higher aspect ratio (AR), AR = h/w_opt_, can achieve higher S_wg_ reaching 0.513 for the TE mode with w = 104 nm and h = 300 nm (AR = 2.88). This behavior is similar for all wavelengths. However, high AR waveguide sensors are more challenging to fabricate and expensive as they need a fine mask and complex lithography system to obtain the small waveguide widths needed.

Moreover, at high AR the waveguide sensitivity is very sensitive to width variations. For example, changing the width by only 20 nm for the TE mode with AR = 2.88 will significantly reduce S_wg_ to lower than 0.15, i.e., 3.4 times reduction. Moreover, this optimum width (w_opt_ = 104 nm) is close to the mode cut-off width (w_cut-off_ = 78 nm) and multimode width (w_MM_ = 110 nm). Figure 2c shows that for high thicknesses with low optimum widths (high AR), TE mode can achieve higher S_wg_ than TM mode, while for lower thicknesses (higher w_opt_ and low AR) TM mode exhibit higher S_wg_ than TE mode. This is because the TE/TM modes have field discontinuity at the core edges in the x/y direction; hence, decreasing the width/thickness will increase the evanescent field’s amount and, hence, sensitivity. Therefore, TM mode is optimum for cheap and easy to fabricate large feature size sensors.

**Figure 3:**
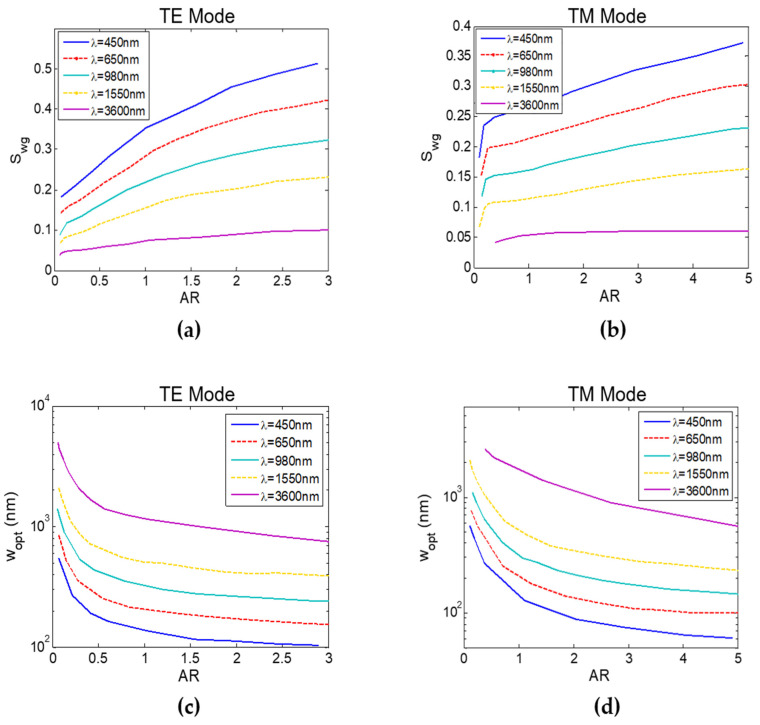
Strip waveguide sensitivity (S_wg_) versus waveguide aspect ratio (AR) for: (**a**) TE mode and (**b**) TM mode at different operating wavelengths. Optimum width (w_opt_) versus waveguide aspect ratio (AR) for: (**c**) TE mode and (**d**) TM mode at different operating wavelengths.

Figure 3 shows the optimum S_wg_ and the optimum width (w_opt_) dependence with the AR (or h) at different wavelengths for the TE and TM mode, respectively. We can see that both the operating wavelength and AR have a significant effect on the obtained S_wg_. In addition, the highest S_wg_ is obtained at the lowest operating wavelength (λ = 450 nm) and it decreases monotonically as the wavelength increases. It is important to note that, scaling the waveguide dimensions with the wavelength does not result in the same waveguide sensitivity. This is mainly due to the unchanged layer’s thickness that changes its refractive index (representing virus attachment). Results also show that TE mode achieves the highest possible S_wg_ at every wavelength.

**Figure 4:**
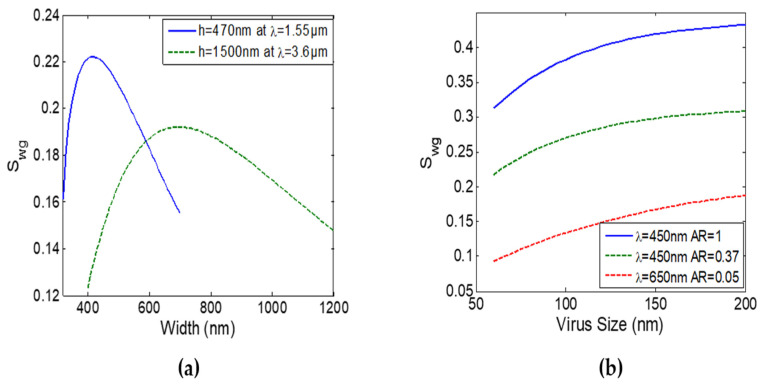
(**a**) Slot waveguide sensitivity S_wg_ versus waveguide width at the optimum waveguide thickness at λ = 1.55 µm and λ = 3.6 µm with w_slot_ = 200 nm. (**b**) Strip waveguide sensitivity S_wg_ of TE mode (solid) and TM mode (dashed) versus virus size (d_vir_) for different optimized designs.

We have also examined Si_3_N_4_ slot waveguides (TE mode only). Slot waveguides sensitivity increase as slot width decrease. Here, we used a slot width (w_slot_) of 200 nm as this is the smallest width that can still allow waveguide functionalization [5,26]. Figure 4a shows the maximum slot waveguide sensitivities at λ = 1.55 µm and λ = 3.6 µm for the TE mode. At λ = 1.55 µm S_wg_ = 0.22 while at λ = 3.6 µm S_wg_ = 0.192. In order to obtain slot mode in the visible range, the slot width should be less than 200 nm hence not suitable for virus detection (functionalization). Consequently, strip waveguides are more suitable for virus detection as they can achieve higher S_wg_ at lower wavelengths leading to much higher FOM. In addition, the functionalization process in a tiny 200 nm slot is challenging. Moreover, strip waveguide offers a simple sensor design, for example, there is no need for a strip to slot mode converter.

While all the previous results are obtained using a virus layer thickness h_vir_ of 80 nm, we also tested the optimized waveguides for different virus diameters (i.e., different h_vir_) from 60 nm to 200 nm. Figure 4b shows that the waveguide sensitivity increases with the virus size. It is important to note that, the optimum waveguide dimensions do not change significantly from the one obtained for h_vir_ = 80 nm, by changing the virus size. Hence, the same waveguide can be used for a range of viruses with different diameters. Moreover, as mentioned above, our analysis is independent of the virus refractive index. Thus, this work can be considered as a universal virus detection method using the SiNOI waveguide platform where the optimized waveguides are well suited for various viruses with different sizes and refractive indices.

**Figure 5:**
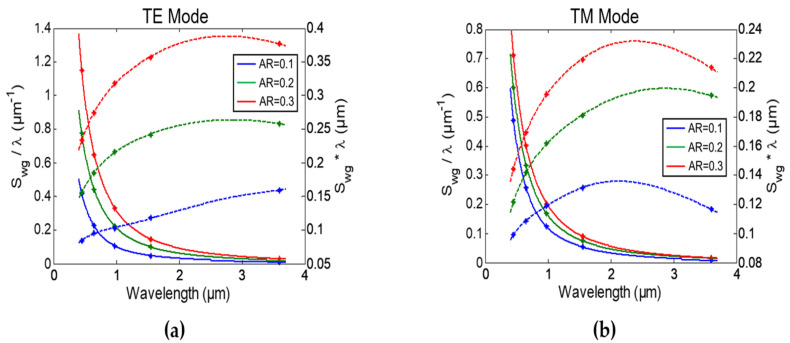
Fitted S_wg_/λ (solid line) and S_wg_ × λ (dashed line) of strip waveguide versus wavelength at different AR with (*) representing the simulated results for: (**a**) TE mode and (**b**) TM mode.

While waveguide sensitivity is an important parameter, the RI optical sensors’ overall performance is determined by the FOM. In both interferometers and resonators, the sensitivity (S) is proportional to S_wg_ × λ. However, the FOM is proportional to S_wg_/λ because the FWHM is proportional to λ^2^. Hence, operating the sensor at lower wavelengths will achieve the highest performance as S_wg_ increases and λ decreases, maximizing the FOM. Figure 5 shows fitted curves of both S_wg_ × λ and S_wg_/λ terms versus wavelength for different AR. It can be seen that, FOM increased around 8 times from NIR (λ = 1.55 µm) to visible (λ = 450 nm) wavelength for both TE and TM modes while the sensitivity decreased only 1.4 times. Moreover, working in the visible wavelength range has another advantage for biosensing as in this range the losses due to water absorption are minimized. From more than 200 dB/cm mode loss at λ = 3.6 µm to less than 3 × 10^−3^ dB/cm in the visible range.

## 3. Virus Sensors Design

In this section, different MZI sensors have been designed to convert the change in the waveguide’s effective index to a sensible quantity for virus detection. The Si_3_N_4_ waveguide surface will be functionalized with the virus antibodies. In this case, when the sensor is exposed to the sample the targeted virus will be selectively captured by the waveguide. Virus binding will change the refractive index of the 80 nm layer covering the waveguide core. Accordingly, a wavelength shift (Δλ) in the transmission spectrum of the MZI will occur, from which the virus concentration can then be determined.

For an MZI device with power evenly divided to its arms, the transmission spectrum can be derived to be [27]:

(2) $$T=\cos^{2}(\frac{\Delta \varphi }{2})$$

(3) withΔφ=2πλ(neff,sensL−sensneff,refLref)

where Δφ the phase difference of the MZI arms; n_eff,sens_, n_eff,ref_ and L_sens_, L_ref_ are the waveguide mode effective index and length of the sensing and reference arms of the MZI sensor, respectively.

From which we can get the peak wavelengths as:

(4) λpeak=1q(neff,sensL−sensneff,refLref)

where q is an integer.

Accordingly, the free spectral range (FSR), full-width half maximum (FWHM), sensitivity (S) and FOM of the MZI sensor can be derived as follows [27]:

(5) $$\mathrm{FSR}=\frac{\lambda^{2}}{n_{\mathrm{eff},\mathrm{sens}}L_{\mathrm{sens}}-n_{\mathrm{eff},\mathrm{ref}}L_{\mathrm{ref}}}$$

(6) $$\mathrm{FWHM}=\frac{\sqrt{2}\mathrm{FSR}}{\pi }$$

(7) $$S=\frac{d\lambda_{\mathrm{peak}}}{\mathrm{dn}}=\frac{\lambda S_{wg}L_{\mathrm{sens}}}{n_{\mathrm{eff},\mathrm{sens}}L_{\mathrm{sens}}-n_{\mathrm{eff},\mathrm{ref}}L_{\mathrm{ref}}}$$

(8) $$\mathrm{FOM}=\frac{S}{\mathrm{FWHM}}=\frac{\pi S_{\mathrm{wg}}L_{\mathrm{sens}}}{\sqrt{2}\lambda }$$

FOM is the main performance parameter of any RI sensor as it determines the minimum detectable refractive index change. Table 1 shows the dimensions and FOM of different symmetric MZI (s-MZI) sensors designs, L_sens_ = L_ref_ = L_MZI_, at different wavelengths with AR≈1, L_MZI_ = 500 µm, and ideal y-junction for comparison. The optimized strip waveguides from the previous analysis with the virus layer around the core are used as the sensing arm with width w_sens_. Oxide-capped waveguides are used as the reference arm with width w_ref_. These results are obtained using an FDE solver to determine n_eff_(λ) of the sensing and reference waveguides. Next, an integrated photonics circuit simulator [28] is used to determine T(λ) at different n_layer_ (virus concentration) from which S, FWHM, and FOM are then calculated. Results exhibit the same response as Figure 5 with around 8 times greater FOM at the lower (blue) wavelength. Table 1 also shows FOM at λ = 450 nm for different AR, reaching a maximum of 1231 RIU^−1^ at AR = 2.88. As mentioned before, at higher waveguide widths, TM mode was used as it can reach higher S_wg_ than TE mode, see Figure 2c. Note that, MIR range was discarded due to its low S_wg_ and high (water absorption) losses.

Although small waveguide dimensions in the visible range exhibit high sensing performance, the fabrication of such waveguides requires complex and expensive lithography systems such as electron beam or deep UV lithography. Hence, we want to determine a sensor’s performance with a feature size above 1 µm, which will allow for easy and cheap fabrication. While lower wavelengths exhibit higher performance, the blue wavelength has almost zero sensitivity for small AR waveguides with w_opt_ > 1 µm. Hence, we choose to compare two designs both with TM mode. The first design is operating at low (red) wavelength λ = 650 nm exhibiting S_wg_ of 0.115, and the second is operating at a higher wavelength at λ = 980 nm but demonstrating a slightly higher S_wg_ of 0.13. Table 2 shows the dimensions and the FOM of both MZI sensor designs. We can see that the first design operating at a lower (red) wavelength with AR = 0.05 has a higher FOM of 158 RIU^−1^ even if it exhibits slightly lower S_wg_.

The minimum detectable index change of the virus layer can be calculated from [29] as Δn_min_ = 1/FOM. These values can then be converted to minimum detectable virus coverage r_min_ using Equation (1). Table 3 shows Δn_min_ and r_min_ for the MZI sensors designs at the blue wavelength with different AR and the design at red wavelength optimized for large dimensions (AR = 0.05) with L_MZI_ = 500 µm. Note that, lower virus concentrations (coverage r) can be detected by increasing the FOM by increasing the MZI sensor length as given in Equation (8).

It is important to note that, silicon nitride waveguides with film thickness greater than 300 nm suffer significant stress, and different techniques are used to overcome this problem [30,31,32]. However, our analysis shows that thin silicon nitride waveguides, with h < 300 nm, in the visible range are of better sensing performance. In this case, such stress is reduced and a homogeneous index and thickness can be obtained using low-pressure chemical vapor deposition (LP-CVD) [32].

**Figure 6:**
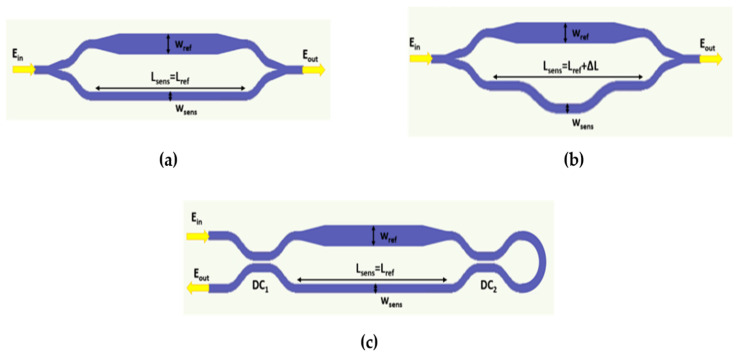
Schematic of MZI sensor configurations: (**a**) s-MZI, (**b**) a-MZI, and (**c**) LT-MZI.

Finally, different MZI configurations were studied and compared for sensing, namely, symmetric MZI (s-MZI), asymmetric MZI (a-MZI), and loop-terminated MZI (LT-MZI) shown in Figure 6. The simulated results of the different configurations are summarized in Table 4 for the design of TM mode with w_sens_ = 270 nm, h = 100 nm, and w_ref_ = 300 nm at λ = 450 nm and L_sens_ = 500 µm. While s-MZI (L_sens_ = L_ref_) sensitivity is determined only by its waveguide structures, i.e. Δn_eff_ = n_eff,sens_−n_eff,ref_, a-MZI (L_sens_ = L_ref_ + ΔL) sensitivity can be engineered using ΔL = L_sens_−L_ref_, according to Equation (7). However, both structures will exhibit almost the same FOM for the same L_sens_. On the other hand, LT-MZI is a recently proposed design [33] that consists of a conventional MZI with a loop connecting the output directional coupler arms, reflecting the wave back to the interferometer. For the same waveguide structure and L_sens_, LT-MZI will exhibit the same sensitivity with the conventional MZI while the FWHM will reduce to half resulting in twice the FOM. The LT-MZI directional couplers are also assumed to be ideal 3-dB couplers. The asymmetric LT-MZI can also be used to control the sensitivity using ΔL as in the a-MZI case.

## 4. Edge Fiber Coupling of Designed Sensors

Recently, many efforts have been done to couple light from fiber to Si_3_N_4_ platform in the NIR range (around 1.55 µm) [34,35,36], reaching a measured coupling efficiency as low as −1.75 dB [34], using a bottom multilayer reflector and an apodized grating coupler. However, few works have been published for coupling in the visible wavelength range [37,38,39].

**Figure 7:**
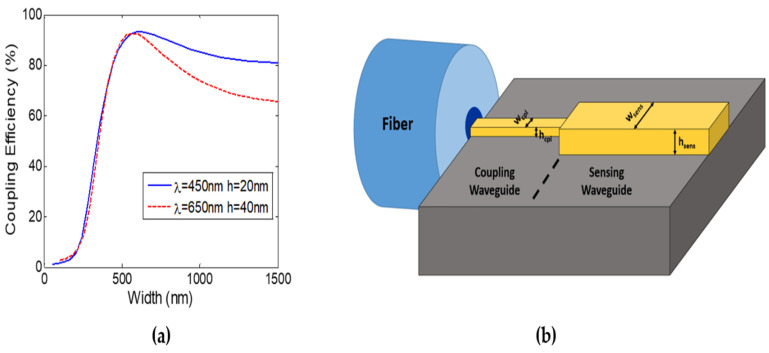
(**a**) Fiber to strip waveguide coupling efficiency versus waveguide width at λ = 450 nm and λ = 650 nm with h = 20 nm and h = 40 nm, respectively. (**b**) Schematic of two-step fiber edge coupling.

In this section, we study the coupling from single-mode fibers [40] in the visible region (blue and red) to the silicon nitride chip through edge coupling again using an FDE solver. We focus on the coupling to the TM mode sensors designs mentioned in the previous section, which can achieve high coupling efficiencies and high FOM with waveguide widths larger than 250 nm, see Table 1 and Table 2. Design 1: w_sens_ = 550 nm, h = 70 nm (AR = 0.13) and Design 2: w_sens_ = 270 nm, h = 100 nm (AR = 0.37), both at λ = 450 nm. While Design 3: w_sens_ = 1500 nm, h = 80 nm (AR = 0.05) at λ = 650 nm for a large feature size sensor (w > 1 µm). Figure 7a shows the coupling efficiency at blue and red wavelengths to a waveguide with thickness h = 20 nm and h = 40 nm, respectively. A maximum coupling efficiency of 93% and 92.7% can be achieved from the fiber to the waveguide TM mode at λ = 450 nm with w = 600 nm and at λ = 650 nm with w = 565 nm, respectively. Note that, waveguides with higher thicknesses exhibit significantly lower coupling for w > 250 nm. Hence, there is a mismatch between the waveguide dimensions with optimum fiber coupling (w_cpl_, h_cpl_) and optimum sensing (w_sens_, h_sens_), as shown in Figure 7b. Accordingly, the coupling between these two waveguides was studied and the insertion loss (IL) was determined for the different sensing waveguides. For each design, we optimize the waveguide–waveguide coupling, w_cpl_ × h_cpl_ → w_out_ × h_sens_, by changing the output waveguide width (w_out_), which can then be converted to w_sens_ with significantly low losses using a taper. Hence, for the blue wavelength the IL from the fiber to the optimum coupling waveguide, w_cpl_ = 600 nm, and h_cpl_ = 20 nm, is 0.3 dB. The waveguide–waveguide coupling for the sensing waveguide with h_sens_ = 70 nm (Design 1) shows IL = 3.8 dB with an optimum w_out_ of 2600 nm. While for the sensing waveguide with h_sens_ = 100 nm (Design 2) IL = 4.8 dB at w_out_ = 2700 nm. Hence, the overall fiber coupling loss to the sensing waveguide is 4.1 dB and 5.1 dB, respectively. For the red wavelength (Design 3), with w > 1 µm, the fiber-coupling waveguide IL is 1.37 dB at w_cpl_ = 1030 nm and h_cpl_ = 40 nm. While the waveguide–waveguide coupling exhibits IL = 1.1 dB for w_out_ = 1940 nm to the sensing waveguide with h_sens_ = 80 nm, resulting in an overall coupling loss of 2.47 dB.

These designs exhibit higher coupling efficiencies than most fiber couplers proposed for the Si_3_N_4_ platform at the same wavelength range [37,38,39]. This is mainly due to the different waveguide dimensions, as the optimum waveguides for sensing have small core thickness dimensions; thus, exhibiting a large mode size which leads to better matching with the fiber mode. The recently proposed fiber couplers and our proposed ones are summarized in Table 5.

## 5. Conclusions

We propose a Si_3_N_4_ strip waveguide to be used as the sensing arm in different integrated optical sensors configurations for virus detection. Integrated RI sensors offer fast, cheap, and simple detection when compared with the existing methods such as PCR, which is expensive and involves complex procedures. Our theoretical study shows that the Si_3_N_4_ strip waveguide sensors can achieve high sensitivity, and with surface functionalization, they can detect only a specific virus for high selectivity. Our numerical analysis determines the waveguide dimensions that maximize the sensitivity to the virus layer attached to its surface. The optimum dimensions were determined for different wavelengths from the visible to the MIR and for both fundamental quasi-TE and quasi-TM modes. In addition, we compared the silicon nitride strip waveguide with the slot waveguide. The results show that strip waveguide operating at low wavelengths is the best choice for virus detection. MZI sensors were designed offering a FOM as high as 1231 RIU^−1^ for L_MZI_ = 500 µm at λ = 450 nm. Finally, edge coupling from the fiber to the waveguide sensor was studied, showing only 4.1 dB insertion loss at λ = 450 nm for the MZI design with FOM = 500 RIU^−1^. Our work forms a universal virus detection method using the SiNOI waveguide platform. This is because the optimized waveguides are well suited for various viruses with different sizes, refractive indices, and generally for the detection of different biomolecules using functionalized waveguide surfaces.

## Figures and Tables

**Table 1:** FOM of S-MZI sensors with L_MZI_ = 500 µm.

λ (nm)	AR	Dimensions (nm)	FOM (RIU^−1^)
450	0.13 *	w_sens_ = 550, w_ref_ = 520 and h = 70	501
	0.37 *	w_sens_ = 270, w_ref_ = 300 and h = 100	553
	1	w_sens_ = 138, w_ref_ = 145 and h = 140	812
	2.88	w_sens_ = 104, w_ref_ = 104 and h = 300	1231
650	1.1	w_sens_ = 203, w_ref_ = 230 and h = 220	454
980	1.2	w_sens_ = 300, w_ref_ = 360 and h = 360	244
1550	1	w_sens_ = 512, w_ref_ = 850 and h = 500	100

* Representing TM mode.

**Table 2:** FOM of S-MZI sensors with large feature size using TM mode at LMZI = 500 µm.

λ (nm)	Dimensions (nm)	FOM (RIU^−1^)
650	w_sens_ = 1500, w_ref_ = 1000 and h = 80	158
980	w_sens_ = 1500, w_ref_ = 1100 and h = 160	127

**Table 3:** Δn_min_ and R_min_ of S-MZI sensors with L_MZI_ = 500 µm.

λ (nm)	AR	Δn_min_	r_min_ (%)
450	0.13 *	2.0 × 10^−3^	1.29
	0.37 *	1.8 × 10^−3^	1.16
	1	1.2 × 10^−3^	0.79
	2.88	8.1 × 10^−4^	0.52
650	0.05 *	6.3 × 10^−3^	3.73

* Representing TM mode.

**Table 4:** FOM and S of the TM mode for different MZI sensors configurations with w_sens_ = 270 nm, h = 100 nm, and w_ref_ =300 nm at λ = 450 nm and L_sens_ = 500 µm.

		S (nm/RIU)	FOM (RIU^−1^)
s-MZI		3098	553
a-MZI	ΔL = 30 µm	1316	540
	ΔL = 5 µm	5579	530
LT-MZI		3098	1106

**Table 5:** Comparison of our proposed fiber couplers and recently demonstrated ones, showing the output waveguide (w × h) in each case with (S) and (M) denoting simulated and measured results, respectively.

	λ (nm)	Waveguide (nm)	Technique	Coupling Loss (dB)
[37]	660	700 × 100	Grating	4.2 (M)
[38]	532	350 × 180	Grating	6 (S)
	640	340 × 220	Grating	6.6 (S) 7.5 (M)
[39]	430–648	340 × 135	Edge	8–8.9 (M)
Our Designs	450	550 × 70	Edge with h step	4.1 (S)
		270 × 100	Edge with h step	5.1 (S)
	650	1500 × 80	Edge with h step	2.47 (S)

## Data Availability

Not applicable.
